# Paul Russell: the transcendentalist surgeon of America

**DOI:** 10.3389/frtra.2023.1191149

**Published:** 2023-06-06

**Authors:** Reza Abdi

**Affiliations:** Transplantation Research Center, Brigham and Women's Hospital, Harvard Medical School, Boston, MA, United States

**Keywords:** Russell, surgeon, transplant, thinker, research

## Abstract

I first met Dr. Russell in the Fall of 2000 at the Massachusetts General Hospital (MGH). I entered the Russell-Round-Room which was packed with surgeons and physicians of MGH, among whom there was no shortage of self-esteem. I came across a handsome man, full of vigor and competence, standing still for nearly two hours in the corner of the room near the blackboard. He was remarkably attentive to the questions, for which he had very concise responses. He was soft-spoken with an inviting smile, and had a welcoming, modest air about him. Despite his remarkable academic achievements, he was strikingly unassuming and serene -- features likely ingrained in his very nature.

I first met Dr. Russell in the Fall of 2000 at the Massachusetts General Hospital (MGH). I entered the Russell-Round-Room which was packed with surgeons and physicians of MGH, among whom there was no shortage of self-esteem. I came across a handsome man, full of vigor and competence, standing still for nearly two hours in the corner of the room near the blackboard. He was remarkably attentive to the questions, for which he had very concise responses. He was soft-spoken with an inviting smile, and had a welcoming, modest air about him. Despite his remarkable academic achievements, he was strikingly unassuming and serene—features likely ingrained in his very nature.

Growing up in Iran, where one is infused with the mystical poetry of Rumi and Hafiz, I was struck by the mystic aura of a “thinker” around him. In addition to being an outstanding physician-scientist, his success in mentoring outstanding scientists and building superb programs, all very different features, were indeed indebted to his “thinker” quality which formed the foundation for a superb research environment for all comers with talent. He reminded me of the famous transcendentalist writers of Concord (MA)—Ralph Waldo Emerson and Henry David Thoreau—who were drawn to the mysticism of the East and Hafiz's poetry. Thoreau and Emerson were also, despite being at the pinnacle of their writing careers, better known for their unassailable mysticism with a wider perspective about life. Their simplicity and ingenuity were as remarkable as their intellectual greatness.

Recently, I was privileged to interview Dr. Russell. It was an inspiring and uplifting experience. He was residing at the same place as his old friend, Francis Moore, another giant of American surgical research and a key person leading the efforts to the first successful kidney transplantation in 1954. Moore had passed away long ago. It was Moore who called Dr. Russell one Sunday morning in the mid- 1960s to “say that he had heard on the radio of a patient recently admitted to the MGH who might prove to be a perfect donor of a liver for one of his patients.” A phone call led to the formation of the Boston Interhospital Organ Bank and later, the broader based New England Organ Bank and the establishment of one of the earliest systems of organ sharing for transplantation.

During our interview over Zoom, at age 97 recovering from COVID, he looked straight into the camera for two hours, gracefully poised but solid, sharply focused with his undivided attention. His memory was impeccable, and his responses were again short and to the point, all likely reflecting his lifelong characteristics. More than 20 years had passed since the first time I met him, and his soul was unaltered. He remained that larger-than-life man with vigor, sharp intellect, and above all, kindness.

Dr. Russell was born in 1925 and raised in Chicago, Illinois. It is probably unknown to many Bostonians that, some of their finest surgeons had come from the Mid-west. His father was a self-made man who became a very famous football player in 1913, played quarterback and earned All-Big Ten honors. Dr. Russell's mother, on the other hand, was an avant-garde artist who had a major role in the formation of modern dance in America. Dr. Russell was trained in the labs of two Nobel Laureates– Charles Huggins and Peter Medawar. Medawar was an unknown zoologist who was conducting experiments which were not of interest to many immunologists at that time. Medawar was then 39 years old. Medawar's reply to Dr. Russell's request for a research fellowship position was cheerfully negative that “This is an undergraduate teaching department of zoology”. Medawar's reply which described the types of ongoing projects in his lab, made it even more attractive to Dr. Russell. He used all sorts of means and eventually ended up joining Dr. Medawar's lab (Figure 1).

**Figure 1:**
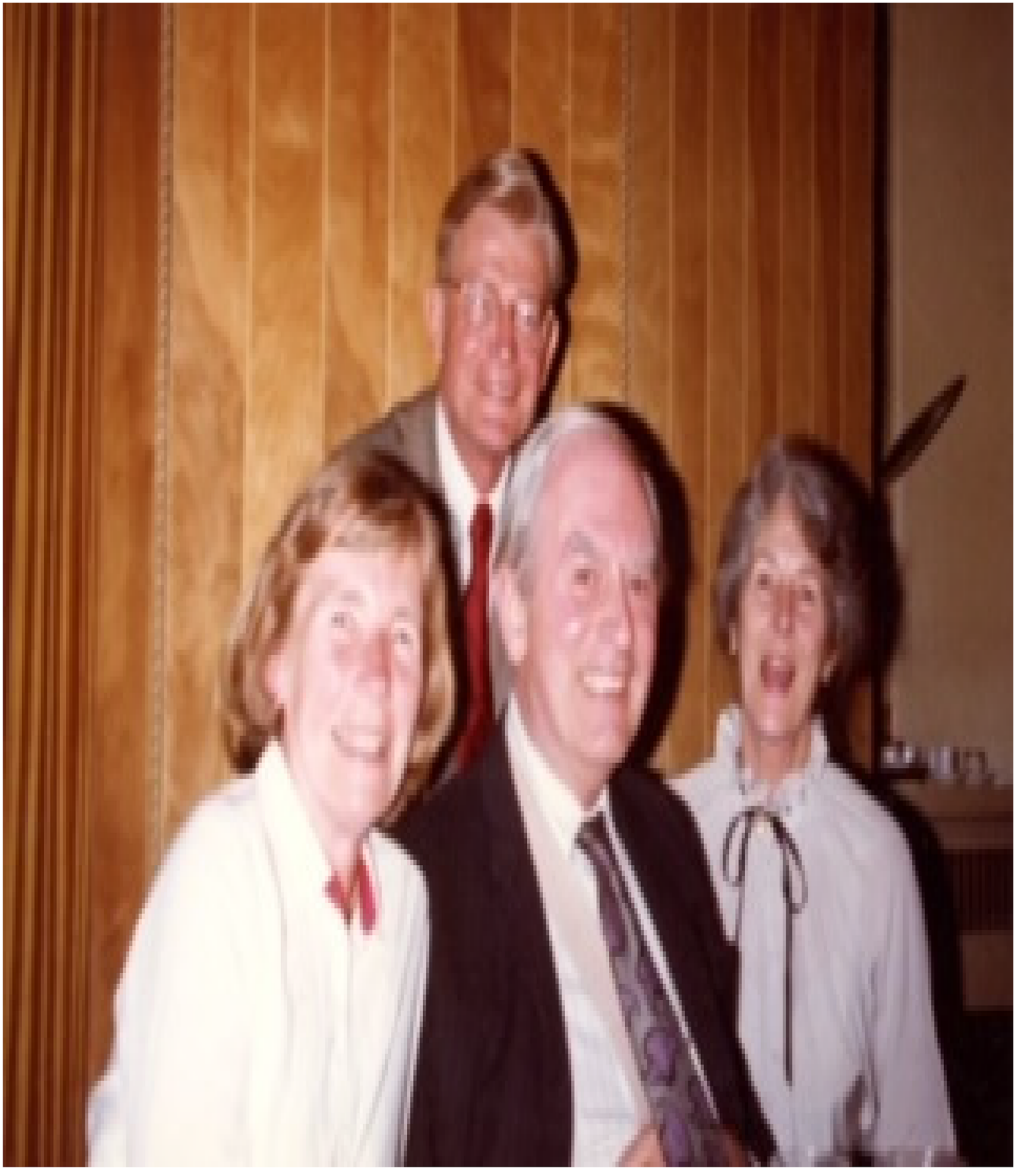
Allene, Paul, and Peter and Jean Medawar.

Most of Dr. Russell's surgery training was at MGH, where he became the Chair of the Department of Surgery and served in that role for nearly a decade. They began kidney transplantation on April 24, 1963 at MGH. His contributions to the field of clinical transplantation are enormous. Notably, he pioneered the concepts of brain death and organ donation. Dr. Russell formed one of the earliest classical academic research labs in the U.S. around preclinical and clinical models of transplantation (1–21). He recruited a scientist, Dr. Henry Winn, who was trained in the lab of the Nobel Laureate George Snell. Dr. Winn was amongst the very first immune-geneticists in pre-clinical organ transplantation research. Dr. Russell's lab attracted numerous key figures early on, not all mentioned here, including Anthony Monaco, Benedict Cosimi, David Sachs, Robert Colvin, Hugh Auchincloss, Megan Sykes and Frank Delmonico. In recognizing 200 years of evolution and innovation in medicine, MGH erected a museum named the Paul S. Russell, MD Museum of Medical History and Innovation (Figure 2).

**Figure 2:**
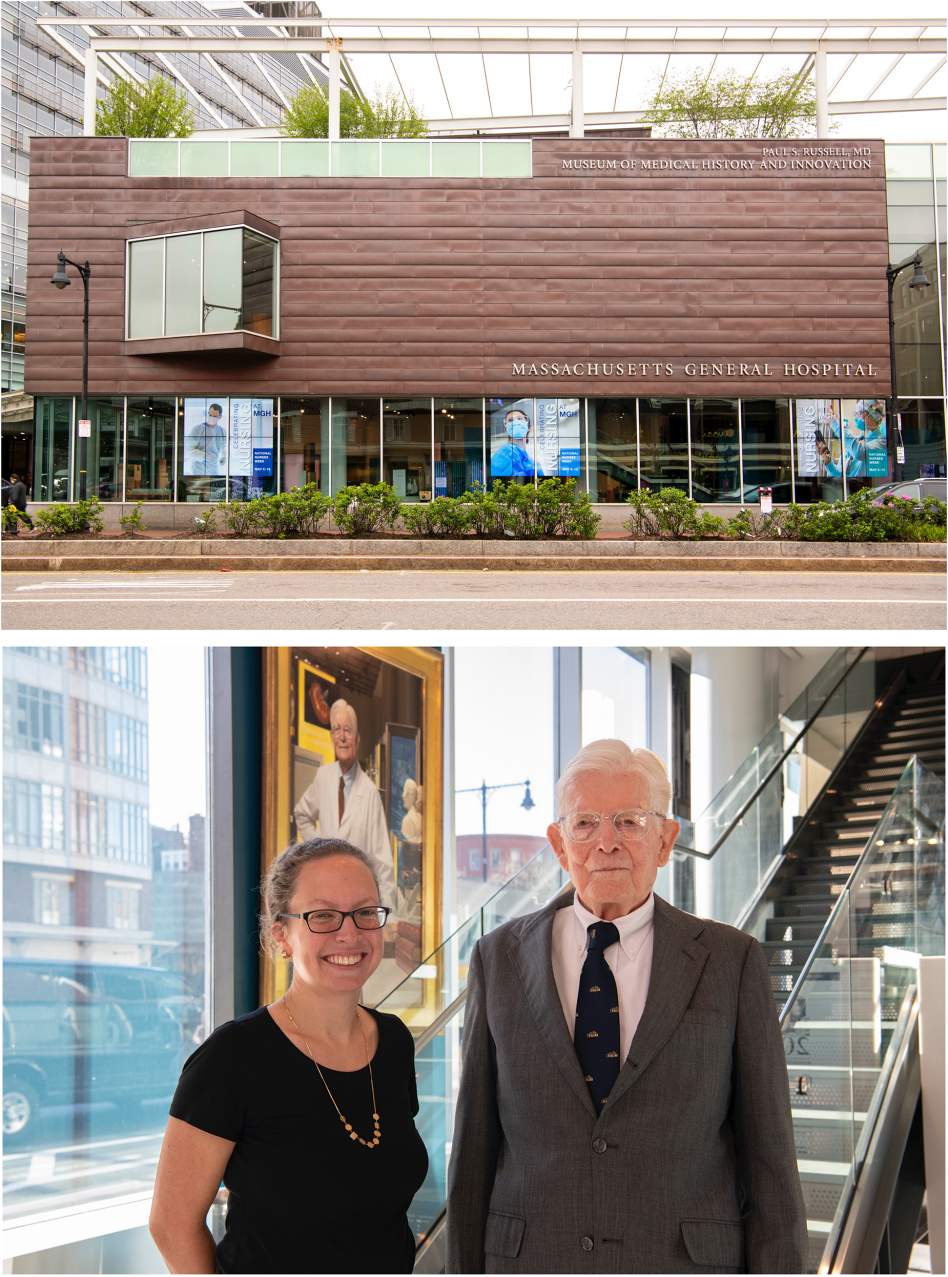
Exterior shot of the museum (Top) and Dr. Russell inside the museum with Sarah Alger, Director of the museum (Bottom). Photo credit for all: MGH Photography Lab.

During our interview, we went over many chapters of his life, over many of his magnificent research accomplishments, but the only time he stopped looking into the camera and gazed upward with his face shining more brightly than usual, was when I asked him about his wife. His answer this time was again brief. He described decades of living with Allene in only a few words, that “she was a wonderful woman” (Figure 3). His response and luminescent expression reminded me of a poem by the Persian Sufi Poet, Rumi: “that if you love someone with your heart and soul, there is no such thing as separation.” If I were to describe Dr. Russell with one word. I would say Optimism. He truly embodies optimism in its fullest sense and inspires others to embrace it as well.

**Figure 3:**
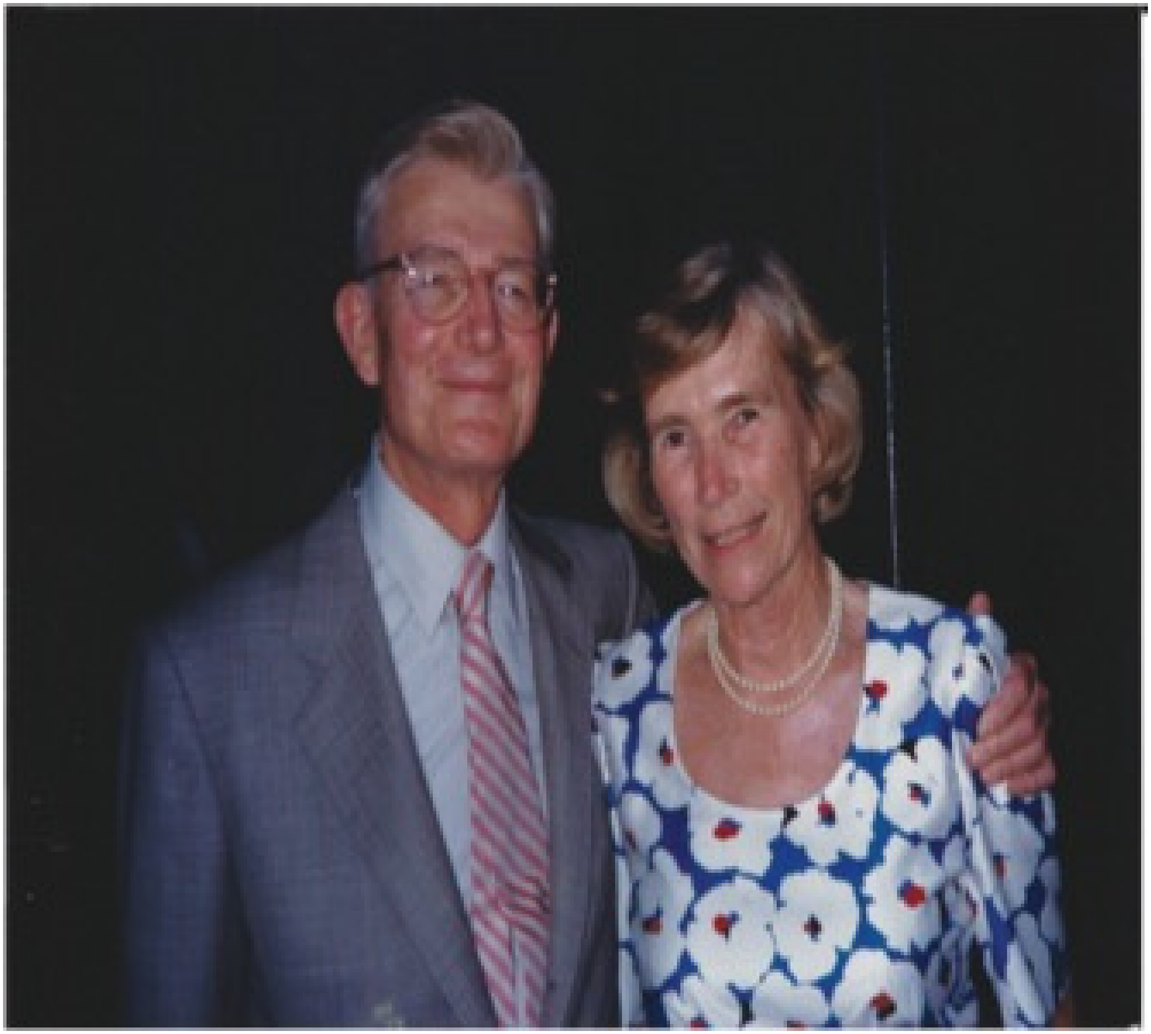
Paul and his wife Allene.

Dr. Russell has seen it all: the unmatched efforts of Abraham Flexner who single-handedly built what then became the foundation of American Medical Research, with the financial support of Rockefeller, at the beginning of the 20th century; the peak of academic research and rise of some of the finest physician-scientists of our time. He has also been witnessing the widespread commercialization of medicine and a tsunami of cultural changes which, one way or other, are impacting American medical leadership and research. While it has been said that the generation of physician-scientists is at the verge of “extinction”, Dr. Russell remains a rarer breed still, a “physician-thinker” that we may never see again (22–24).

## Data Availability

The original contributions presented in the study are included in the article, further inquiries can be directed to the corresponding author.
